# Supplementary material for: Spinochrome D Attenuates Doxorubicin-Induced Cardiomyocyte Death via Improving Glutathione Metabolism and Attenuating Oxidative Stress
Source: Mar Drugs. 2018 Dec 20;17(1):2. doi: 10.3390/md17010002 (PMC6356724; doi:10.3390/md17010002)
Supplement: Supplementary file 1 [file marinedrugs-17-00002-s001.zip › marinedrugs-406270-SI-resubmit 1/marinedrugs-406270-SI-resubmit 1.pdf]

Supplementary Information

Spinochrome D attenuates doxorubicin-induced cardiomyocyte death via improving glutathione metabolism and attenuating oxidative stress

Chang Shin Yoon[1], Hyoung Kyu Kim[1] Natalia P. Mishchenko[2], Elena A. Vasileva[2], Sergey A. Fedoreyev[2], Valentin A. Stonik[2] and Jin Han[1*]

[1]National Research Laboratory for Mitochondrial Signaling, Department of Physiology, College of Medicine, Cardiovascular and Metabolic Disease Center (CMDC), Inje University, Busan 614-735, Korea
[2]G.B. Elyakov Pacific Institute of Bioorganic Chemistry, Far-Eastern Branch of the Russian Academy of Science, Vladivostok, Russia

**Corresponding authors. E-mail: phyhanj@gmail.com*

Figure S1. Spinochrome D (SpD) showed no harmful effect on H9c2 rat cardiac cells. H9c2 rat embryonic cardiomyocytes were treated with 0-200 μM of SpD for 24 h (2×10^4 cells/well, 96-well plates). The cell viability was measured using CCK-8 assay. SpD did not affect on the cell viability of cardiomyocytes. * mark indicates p<0.05 compared with control.

Figure S2. (A) PLS-DA from metabolites of SpD treated AC16 cells. (B) VIP>0.1 metabolites from SpD treated AC16 cells from PLS-DA.

Figure S3. OPLS-DA from metabolites of SpD treated AC16 cells (R2X=0.333; R2Y=0.937; Q2=0.597).

Figure S4. Metabolite sets enrichment overview in SpD treated AC16 cells.

Table S1. Genes and metabolites specified for the assigned pathways.

Figure S5. STRING analysis revealed that metabolism associated proteins forms clusters around mitochondria (marked as red circles).

Figure S6. Spinochrome D (SpD) showed enhanced antioxidant capacity compared with echinochrome A in AC16 and H9c2 cells. (A) SpD and echinochrome A showed no harmful effect on AC16 cells (2×10^4 cells/well, 96-well plate, 24 h). (B) SpD and echinochrome A protected AC16 cells against the cytotoxicity of doxorubicin. (C) SpD showed statistically enhanced antioxidant activity compared with echinochrome A. SpD produced enhanced ATP production in (D) AC16 cells and (E) H9c2 cells compared with echinochrome A. (F) SpD and echinochrome A showed enhanced OCR in AC16 cells. * mark indicates $p<0.05$ compared with control.

Figure S7. SpD did not inhibit the cytotoxicity of doxorubicin. (A) Mitochondrial membrane potential and mitochondrial calcium was visualized using TMRE and rhod-2 staining in MCF-7 cells. Doxorubicin decreased TMRE intensity and increased rhod-2 intensity in dose-dependent manner. The co-treatment of SpD (10 μM) did not affect doxorubicin-induced tendency of fluorescence intensity. (B) The fluorescence was measured using fluorescence spectrometer. SpD co-treatment did not affect the intensity of TMRE, rhod-2, and DCF-DA in MCF-7 cells. (C) TMRE and rhod-2 fluorescence images from HeLa cells with doxorubicin/SpD. (D) The fluorescence measures indicated that SpD did not affect the intensity of TMRE, rhod-2, and DCF-DA in HeLa cells.

Figure S8. SpD inhibited cell migration of cancer cells. 2×10^4 cells of MCF-7 and HeLa were plated in 25-well cell culture plates and scratch wounds were made with 200 μl pipet tips. SpD was treated in 10-200 μM concentrations and incubated for 24 hr.

Figure S9. Total glutathione and ratio of reduced glutathione to oxidized glutathione (GSH/GSSG). (A) Total glutathione ratio (% of control) from SpD treated AC16 cells. (B) Ratio of reduced glutathione (GSH) to oxidized (GSSG) glutathione normalized by total glutathione concentration from control. The GSH/GSSG assay was measured using GSH/GSSG-Glo™ Assay (Promega, WI, USA) according to the product's manual. * and ** mark indicates $p<0.05$, $p<0.01$ compared with control, respectively.

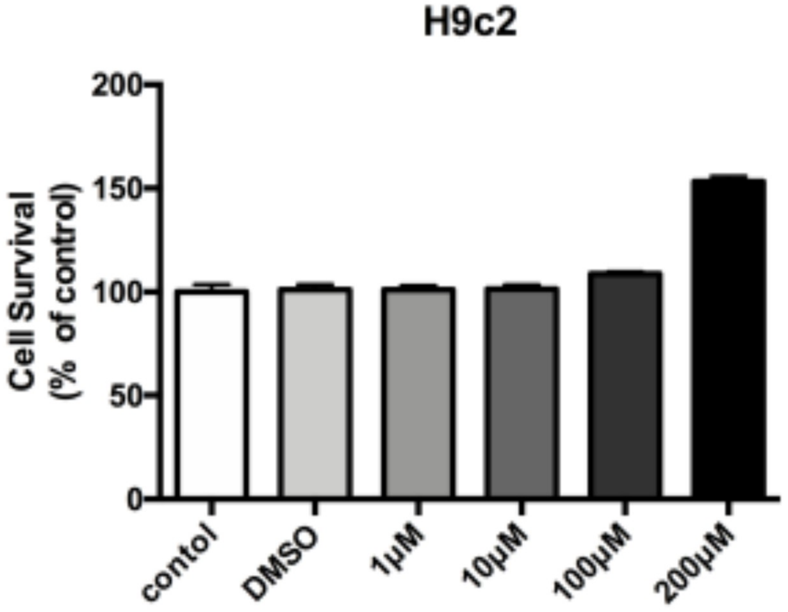

Figure S1. Spinochrome D (SpD) showed no harmful effect on H9c2 rat cardiac cells. H9c2 rat embryonic cardiomyocytes were treated with 0-200 μM of SpD for 24 h (2×10^4 cells/well, 96-well plates). The cell viability was measured using CCK-8 assay. SpD did not affect on the cell viability of cardiomyocytes. * mark indicates p<0.05 compared with control.

A

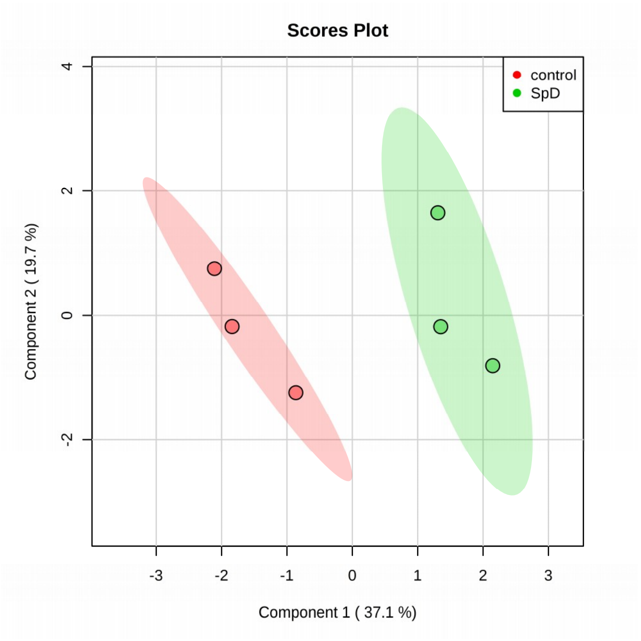

B

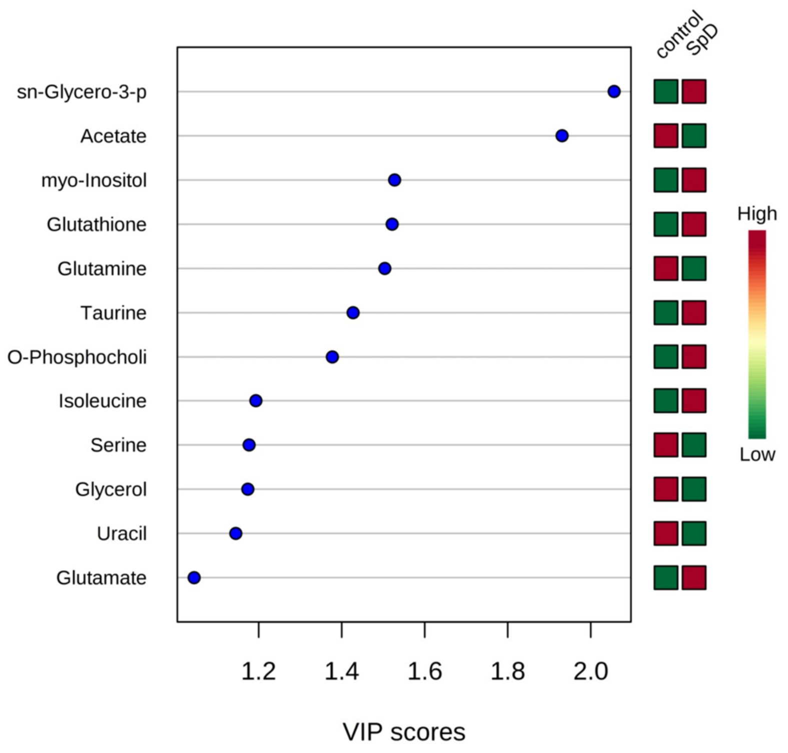

Figure S2. (A) PLS-DA from metabolites of SpD treated AC16 cells. (B) VIP>0.1 metabolites from SpD treated AC16 cells from PLS-DA.

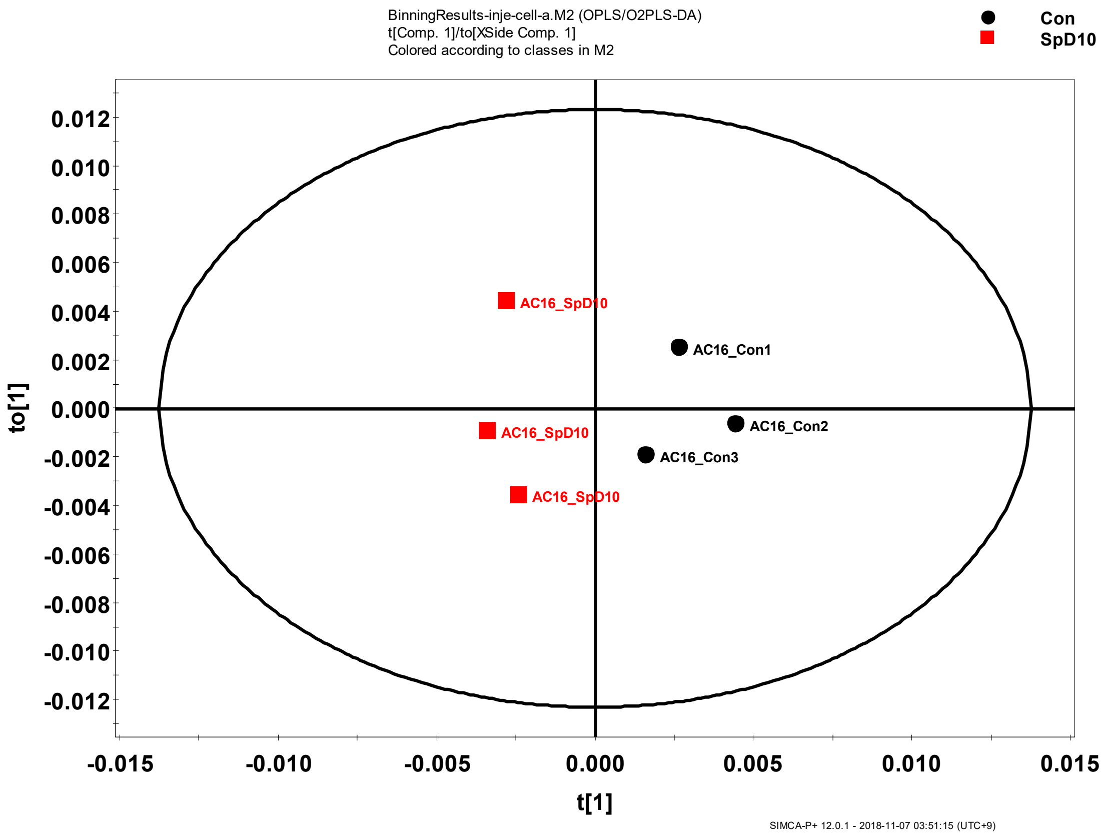

Figure S3 OPLS-DA from metabolites of SpD treated AC16 cells (R2X=0.333; R2Y=0.937; Q2=0.597).

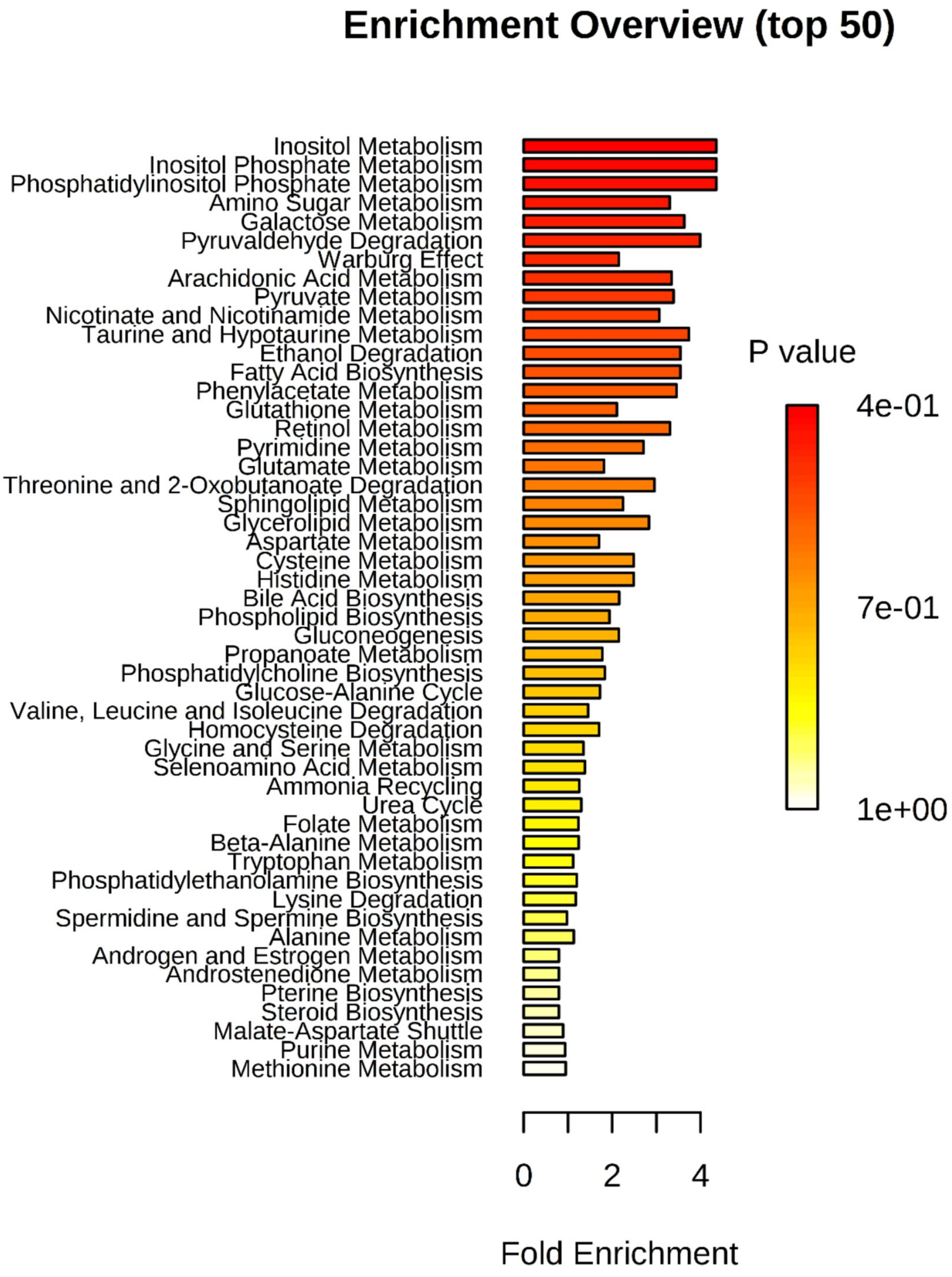

Figure S4. Metabolite sets enrichment overview in SpD treated AC16 cells.

Table S 1. Genes and metabolites specified for the assigned pathways.

Pathway	Gene	Metabolite
Glutathione metabolism	RRM1;MGST1;GSTM2;SRM;GSTK1;GCLM;G6PD;SMS;TXNDC12;GSR;GSS;PGD	glutathione;glutamate
Protein digestion and absorption	COL4A1;COL6A1;COL4A4;COL5A1;COL5A2;DPP4;COL6A2;COL6A3;COL7A1; ATP1B3;COL11A1;COL27A1	acetate;isoleucine;glutamate
Gap junction	MAPK3;MAP2K1;GRB2;PDGFRB;PRKCA;TUBB3;TUBB4A;TUBB4B;TUBB2A;PLCB3; GNAI3;GNAQ;SRC;GNA11;TUBB;GNAS	glutamate
Sulfur metabolism	BPNT1;PAPSS2;PAPSS1;MPST	acetate;taurine
Aminoacyl-tRNA biosynthesis	CARS;QARS;NARS;FARSB;HARS;RARS;TARS;IARS2;YARS;DARS;YARS2;WARS	isoleucine;glutamate
Choline metabolism in cancer	SLC44A2;MAP2K1;GRB2;PDGFRB;PCYT1A;MTOR;AKT2;WASF2;RAC1;RHEB; MAPK3;PRKCA	o-phosphocholine;sn-glycero-3-phosphocholine
Central carbon metabolism in cancer	SLC16A3;MAP2K1;PDGFRB;PDHB;MTOR;TIGAR;AKT2;LDHA;MAPK3;PFKP;G6PD	glutamate;isoleucine
Long-term depression	MAP2K1;PRKCA;GNA11;PLCB3;PPP2R1B;GNAI3;GNAQ;GNAS;MAPK3	glutamate
Long-term potentiation	CALM1;PPP1CA;MAP2K1;PRKCA;CAMK2D;PLCB3;GNAQ;PPP3CA;MAPK3	glutamate

Figure S5. STRING analysis revealed that metabolism associated proteins forms clusters around mitochondria (marked as red circles).

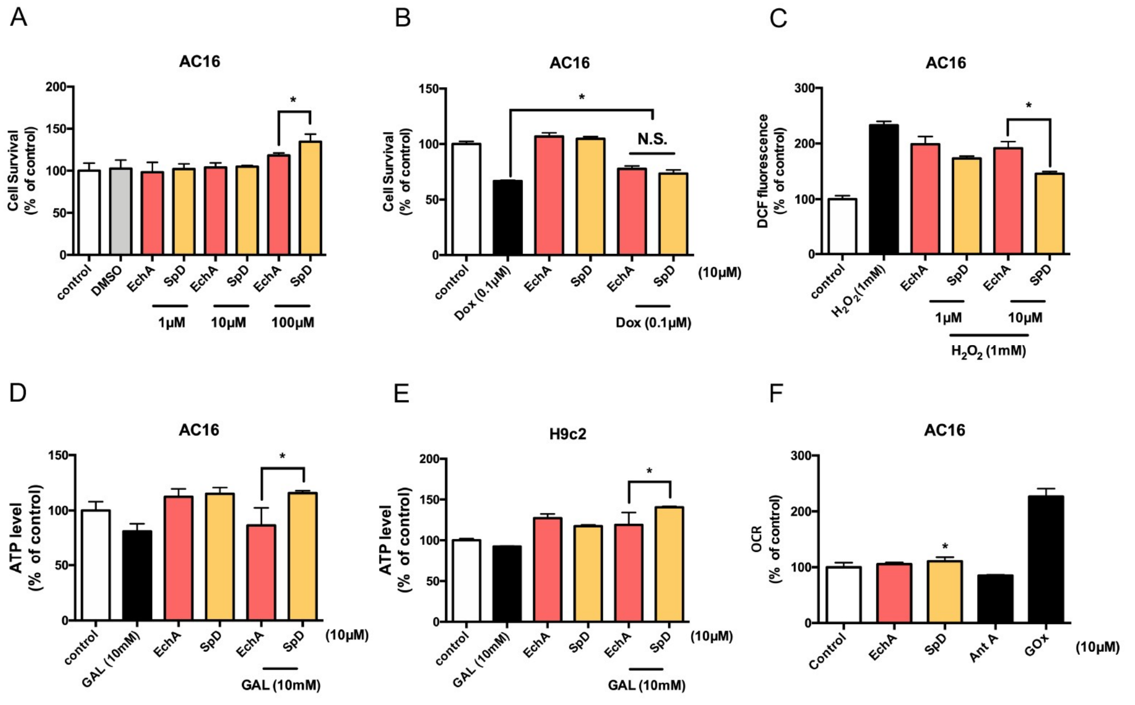

Figure S6. SpD showed enhanced antioxidant capacity compared with echinochrome A in AC16 and H9c2 cells. (A) SpD and echinochrome A showed no harmful effect on AC16 cells (2×10^4 cells/well, 96-well plate, 24 h). (B) SpD and echinochrome A protected AC16 cells against the cytotoxicity of doxorubicin. (C) SpD showed statistically enhanced antioxidant activity compared with echinochrome A. SpD produced enhanced ATP production in (D) AC16 cells and (E) H9c2 cells compared with echinochrome A. (F) SpD and echinochrome A showed enhanced OCR in AC16 cells. * mark indicates $p<0.05$ compared with control.

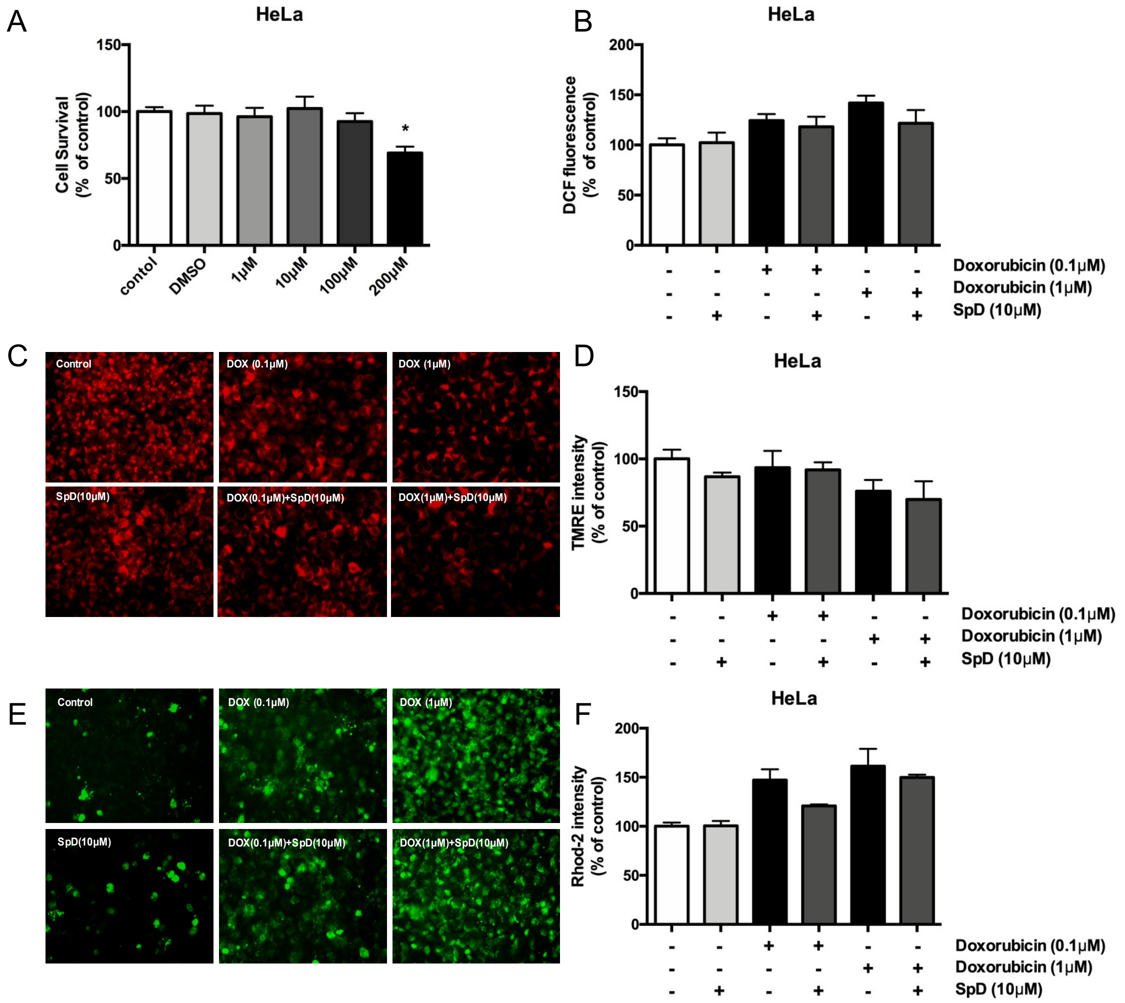

Figure S7. SpD did not inhibit the cytotoxicity of doxorubicin. (A) Mitochondrial membrane potential and mitochondrial calcium was visualized using TMRE and rhod-2 staining in MCF-7 cells. Doxorubicin decreased TMRE intensity and increased rhod-2 intensity in dose-dependent manner. The co-treatment of SpD (10 μM) did not affect doxorubicin-induced tendency of fluorescence intensity. (B) The fluorescence was measured using fluorescence spectrometer. SpD co-treatment did not affect the intensity of TMRE, rhod-2, and DCF-DA in MCF-7 cells. (C) TMRE and rhod-2 fluorescence images from HeLa cells with doxorubicin/SpD. (D) The fluorescence measures indicated that SpD did not affect the intensity of TMRE, rhod-2, and DCF-DA in HeLa cells.

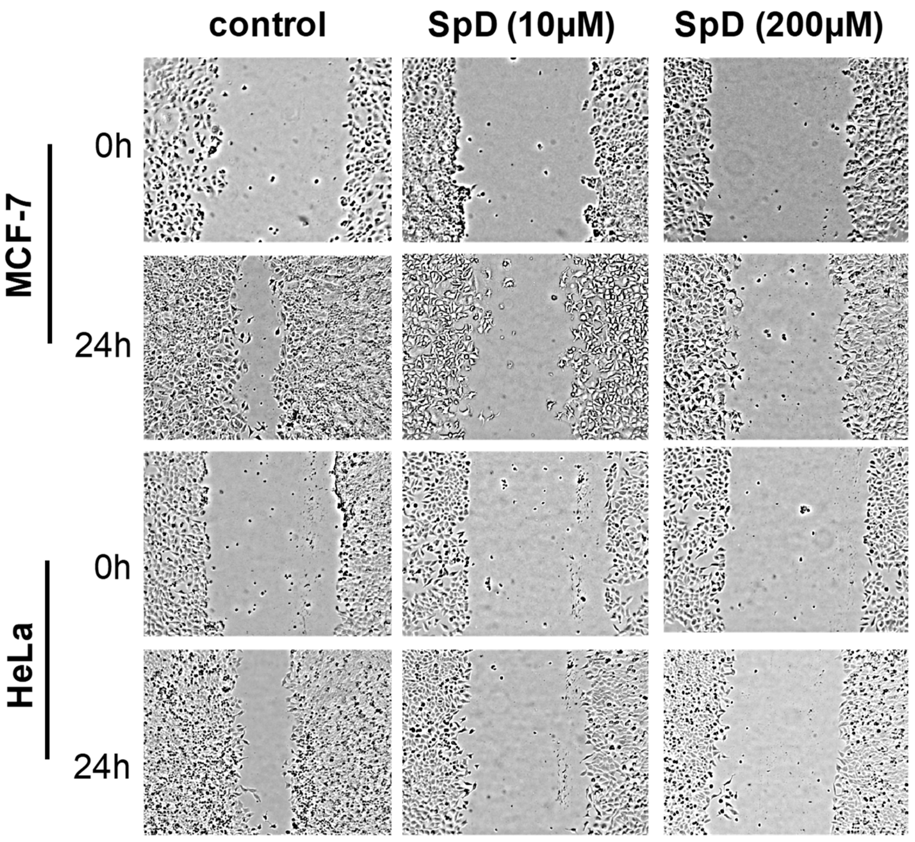

Figure S8. SpD inhibited cell migration of cancer cells. 2×10^4 cells of MCF-7 and HeLa were plated in 25-well cell culture plates and scratch wounds were made with 200 µl pipet tips. SpD was treated in 10-200 µM concentrations and incubated for 24 hr.

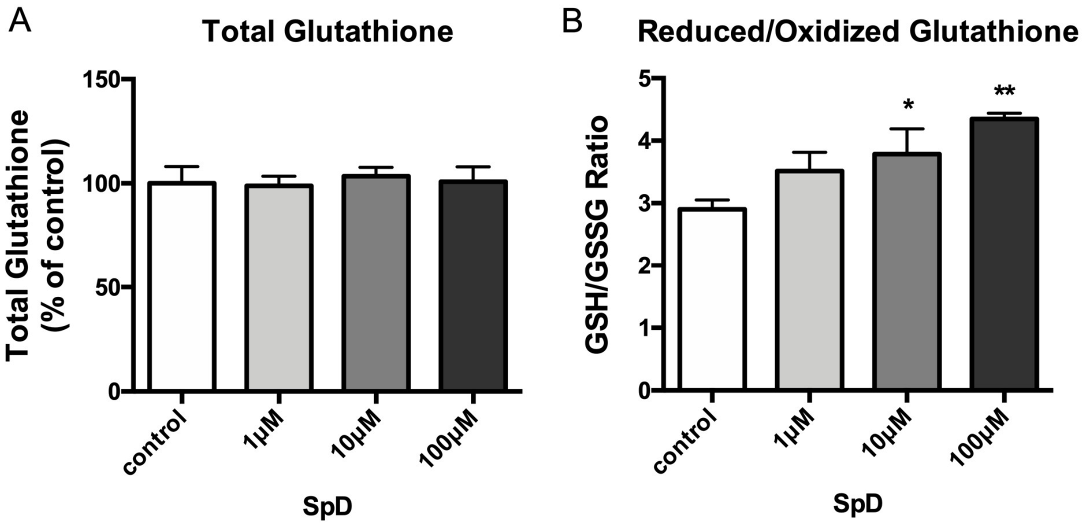

Figure S9. Total glutathione and ratio of reduced glutathione to oxidized glutathione (GSH/GSSG). (A) Total glutathione ratio (% of control) from SpD treated AC16 cells. (B) Ratio of reduced glutathione (GSH) to oxidized (GSSG) glutathione normalized by total glutathione concentration from control. The GSH/GSSG assay was measured using GSH/GSSG-Glo^{TM} Assay (Promega, WI, USA) according to the product's manual. * and ** mark indicates $p<0.05$, $p<0.01$ compared with control, respectively.
